# Are CD4^+^ T-Cell Counts Associated with *Pneumocystis jirovecii* Detection in Hospitalized Patients with Liver Disease? A Retrospective Exploratory Pilot Analysis

**DOI:** 10.3390/livers6030040

**Published:** 2026-05-09

**Authors:** Antonios Katsounas, Amer Nashtar, Jasmin Weninger, Michael Steckstor, Despoina Koulenti, Joshua D. Nosanchuk, Mustafa Özcürümez, Ali Canbay, Peter M. Rath

**Affiliations:** 1 Ruhr University Bochum, Knappschaft Kliniken University Hospital Bochum, Department of Medicine, 44892 Bochum, Germany; 2 Department of Critical Care Medicine, King’s College Hospital NHS Foundation Trust, London SE5 9RS, UK; 3 Departments of Medicine (Division of Infectious Diseases) and Microbiology and Immunology, Albert Einstein College of Medicine, Bronx, New York, NY 10461, USA; 4 Institute of Medical Microbiology, University Hospital Essen, University of Duisburg-Essen, 45122 Essen, Germany

**Keywords:** cirrhosis-associated immunodeficiency, CD4^+^ T-cell, *Pneumocystis jirovecii*, opportunistic infection

## Abstract

**Background:**

Advanced cirrhosis induces profound CD4^+^ T-cell depletion through splenic sequestration and immune dysregulation. *Pneumocystis jirovecii* pneumonia risk thresholds remain undefined in non-HIV immunocompromised populations, necessitating investigation in cirrhotic patients.

**Objectives:**

To investigate the potential association between peripheral CD4^+^ T-cell counts and opportunistic infections (OI)—specifically *Pneumocystis jirovecii* (PJ)—in hospitalized patients with liver disease, and to characterize clinical outcomes across immunological risk strata.

**Methods:**

We retrospectively analyzed 455 adults hospitalized in a single institution with hepatic disorders. CD4^+^ T-cell counts were available in 227/455 patients. Among these, 22 patients met predefined immunological risk criteria (CD4^+^ < 500/μL and/or HIV positivity) and were classified into three immunological risk clusters (IRCs): IRC-A (HIV−, CD4^+^ < 200/μL; n = 9), IRC-B (HIV+, CD4^+^ < 200/μL; n = 7), and IRC-C (HIV−, CD4^+^ 200–499/μL; n = 6). PJ PCR testing was evaluated when available.

**Results:**

Among 455 patients, in-hospital mortality was 103/455 (22.6%). Of the 22 immunologically at risk patients, 15/22 had cirrhosis. PJ PCR was performed in 8/22 patients (IRC-A: 2; IRC-B: 4; IRC-C: 2), with 3/8 positive (37.5%): IRC-A (1/2), IRC-B (1/4), IRC-C (1/2). Ct values ranged from 27.0 to 31.7. Two PJ-PCR–positive cirrhotic patients (IRC-A: n = 1; IRC-C: n = 1) survived without specific anti-PJ therapy; one HIV-positive patient (IRC-B) received trimethoprim-sulfamethoxazole and survived. In-hospital mortality was 5/9 (55.6%) in IRC-A, 2/7 (28.6%) in IRC-B, and 3/6 (50.0%) in IRC-C; none of the deaths were attributable to PJ pneumonia.

**Conclusions:**

Severe CD4^+^ T-cell depletion (<200/μL) was common among cirrhotic patients and associated with PJ detection (3/8 tested), but not with PJ-related mortality (0/3). Mortality was primarily driven by hepatic decompensation and bacterial infections. CD4^+^ assessment may improve risk stratification in cirrhosis; however, prospective, multicenter studies are warranted to validate these findings and to evaluate CD4-guided strategies for the prevention of opportunistic infections.

## 1. Introduction

Patients with acquired immunodeficiency syndrome (AIDS) due to human immunodeficiency virus (HIV) infection, as well as those undergoing immunosuppressive therapy for underlying autoimmune diseases (AID), solid organ transplantation (SOT), or autologous/allogeneic hematopoietic stem cell transplantation (HSCT), are at significant risk for developing opportunistic infections (OI) [1,2]. OI are frequently caused by, for example, cytomegalovirus (CMV), *Toxoplasma gondii* (TG), and *Pneumocystis jirovecii* (PJ) [3–6]. Current research highlights the pivotal role of CD4^+^ T-cells in controlling and resolving these infections. CD4^+^ T-cells facilitate immune responses against CMV, TG, and PJ pneumonia (PJP) by supporting CD8^+^ T-cell and B-cell activation, thereby enabling sustained, long-term immunity [7].

It is widely recognized that both the underlying disease and therapeutic interventions influence the degree of immunosuppression [8,9]. The European Association for the Study of the Liver (EASL)—among other leading expert committees—has acknowledged cirrhosis as an immunocompromised state [10,11]. Cirrhosis-associated immune dysfunction (CAID), encompassing low- and high-grade systemic inflammation phenotypes, reflects dynamic immune impairment and contributes to liver disease progression [12]. Notably, interpretation of absolute CD4^+^ T-cell counts in cirrhosis differs from HIV, as splenic sequestration, hemodilution, and systemic inflammation frequently lower CD4^+^ values independent of cellular immune competence [13]. With over 10 million prevalent cases of decompensated liver disease and more than 110 million living with compensated chronic liver disease globally, cirrhosis is considered a major immunodeficiency condition with significant public health implications [11].

Despite these insights, robust guidelines for OI risk stratification in patients with liver cirrhosis are lacking [14]. Established OI guidelines for HIV/AIDS [3–5], SOT [6], AID [15], or HSCT [16] emphasize serologic and molecular screening for TG and PJ, as well as monitoring absolute and relative CD4^+^ and CD8^+^ T-cell counts [3–5]. However, comparable recommendations for cirrhosis are absent. To date, only a few studies have reported on PJ colonization or infection rates in advanced liver disease, with low prevalence data before liver transplantation (LTx, 4/297; 1.4%) or after LTx (6/1329; 0,45% or 7/343; 2%) [17–19]. Given these low rates, clinically manifest PJP in cirrhotics without additional risk factors is expected to be rare, and small single-centre studies are unlikely to capture incident cases. Beyond case reports and selected entities like autoimmune hepatitis requiring immunosuppression, no systematic evaluation of PJ detection in patients with liver cirrhosis has been performed [20,21]. In particular, the potential role of absolute CD4^+^ (and CD8^+^) T-cell counts for stratifying the risk of PJ—defined by polymerase chain reaction (PCR) positivity—in cirrhotic patients remains widely unexplored.

This retrospective exploratory pilot analysis primarily aims to examine the association between peripheral CD4^+^ (and CD8^+^; shown only in Supplementary Information S1: Table S1) T-cell counts and detection of PJ—in hospitalized patients with liver disease, and to evaluate its potential implications for clinical outcomes.

## 2. Materials and Methods

This study was conducted at the University Hospital Essen, an LTx center, and included all patients admitted to the regular care ward (RCW), intermediate care unit (ImCU), or intensive care unit (ICU) of the Department of Gastroenterology and Hepatology between 1 May 2015, and 31 August 2016.

Patients were eligible for inclusion if their medical records were associated with at least one of the relevant International Classification of Diseases, 10th Revision, German Modification (ICD-10-GM) codes ranging from K70 to K77 (https://www.icd-code.de/icd/code/K70-K77.html, accessed on 13 July 2017). CD4^+^ and CD8^+^ T-cell counts, along with other immune markers, were not routinely assessed in patients with liver disease unless there was diagnostic uncertainty or the patient was enrolled in another trial that included these markers. Data on immune markers were retrospectively collected from measurements made during clinical practice or as part of the other trial. This study and the ancillary trial were approved by the institutional review board (IRB) of the University of Essen (protocol nos. 18–8482-BO and 18–8184-BO).

**Figure 1.**
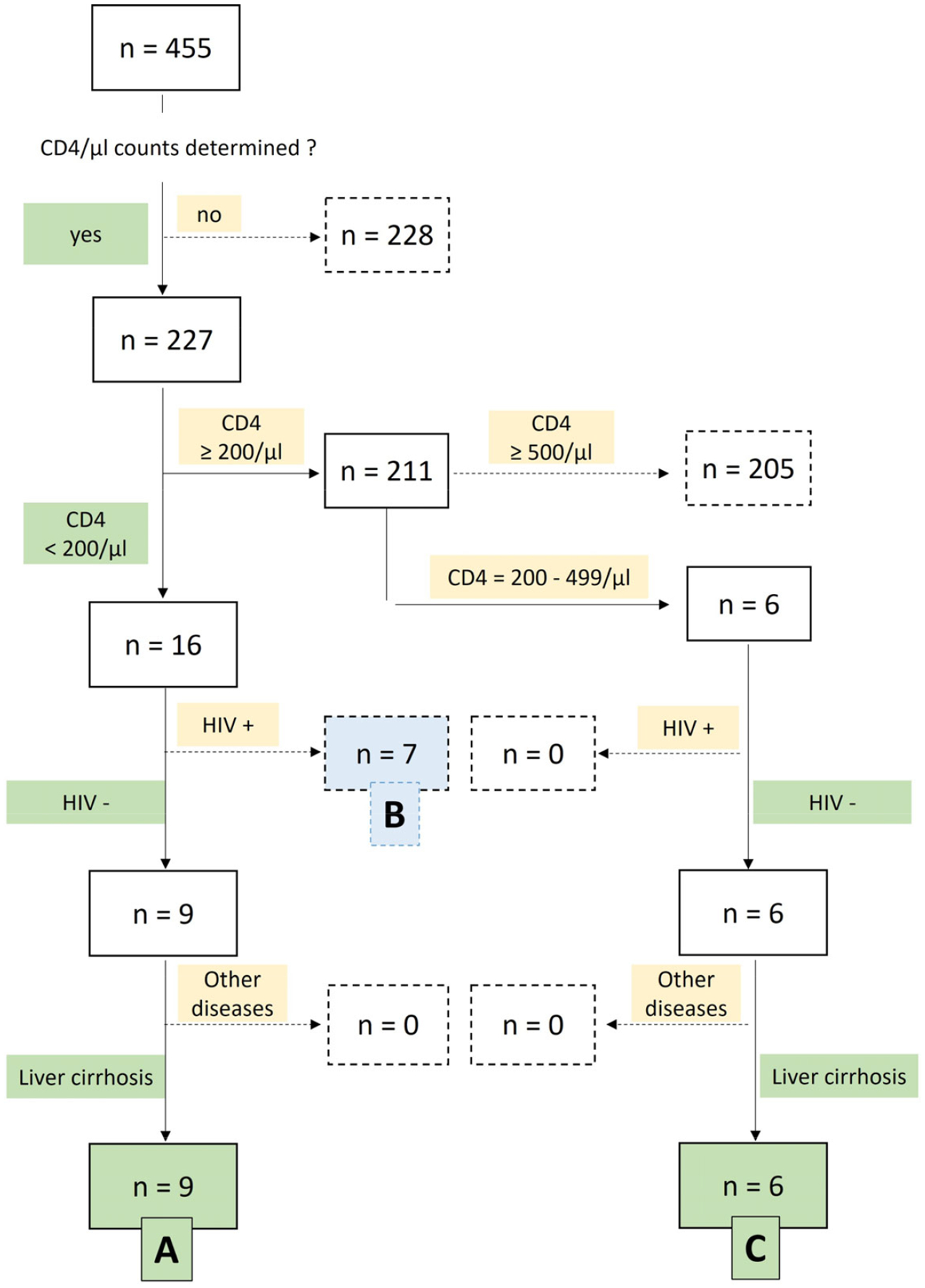
Flowchart for patient classification into IRCs A, B, and C based on CD4^+^ T-cell counts, HIV status, and comorbidities. IRC: Immunological Risk Cluster. HIV: Human Immunodeficiency Virus.

CD4^+^ T-cell counts reflected the immunological status at admission and were obtained before any PJ PCR testing. Patients were stratified at admission according to their CD4^+^ T-cell counts: either <200/μL or ≥200/μL. Further stratification was performed using additional criteria, including CD4^+^ T-cell counts between 200–499/μL and HIV serostatus (positive vs. negative), resulting in three patient immunological risk clusters (IRCs): A, B, and C (Figure 1). For analytical clarity, HIV-positive patients with CD4^+^ <200/μL (IRC-B) were evaluated as a separate comparator group, not combined with cirrhotic patients, to avoid conflating HIV-related immunodeficiency with CAID.

In the next step, we screened patients with liver cirrhosis for available molecular testing results: specifically, PCR-based diagnostics for PJ (Supplementary Information S2). These tests were performed either as part of routine clinical evaluation or in the context of standardized pre-LTx assessments. All available results were extracted and integrated into the datasets of the defined IRCs. For a subset of patients, serum samples were tested for (1→3)-β-D-glucan (BDG) using the Fungitell^®^ assay (Associates of Cape Cod, East Falmouth, MA, USA, and Supplementary Information S1: Tables S2a,b, S3a,b and S4a,b). The analysis also includes additional immunological parameters, such as B cells (CD19+), cytotoxic CD8+ T-cells, natural killer (NK) cells (CD3-, CD16/56+), and CD3+, HLA-DR+ cells, indicative of T-cell activation (Supplementary Information S1: Table S1).

## 3. Results

### 3.1. Levels of Hospital Care and In-Hospital Mortality

This retrospective analysis included 455 hospitalized patients (264 male, 191 female) with various types of hepatic disorders, identified by at least one ICD-10-GM code from the K70.–K77. range in their medical records. The mean age at admission was 65.3 years, and the median hospital stay was 18 days. The overall in-hospital mortality (IHM) across all Levels of required Hospital Care (LHC): RCW, ImCU, ICU) was 22.6% (Table 1).

### 3.2. Immunological Risk Stratification

CD4^+^ T-cell counts were available in 227 patients (49.9%). Based on CD4^+^ levels, HIV status, and the presence of liver disease, a total of 22 patients were classified into three predefined IRCs: A, B, or C, as summarized in Figure 1.

- IRC A (n = 9): HIV-negative patients with liver cirrhosis and CD4^+^ < 200/μL. This group showed the highest IHM at 55.6%.
- IRC B (n = 7): HIV-positive patients with CD4^+^ < 200/μL, showing mildly elevated yet nonspecific transaminase activity and imaging findings consistent with nonalcoholic fatty liver disease (NAFLD)—now termed metabolic dysfunction–associated steatotic liver disease (MASLD; ICD-10-GM: K76.0). There was no evidence of liver cirrhosis in this group. Their IHM was 28.6%.
- IRC C (n = 6): HIV-negative patients with liver cirrhosis and CD4^+^ between 200–499/μL, showing an IHM of 50%.

In total, 22 patients had CD4^+^ counts <500/μL, qualifying as being at risk for OI under established guidelines. Of these, 15/22 (68%) had liver cirrhosis. Within the high-risk subgroup (CD4^+^ < 200/μL, IRCs A and B), 9/16 patients (56%) had cirrhosis and 7/16 (44%) were HIV-positive. The remaining 6/22 (27%), all with cirrhosis and CD4^+^ 200–499/μL, represented the moderate-risk group (IRC C).

Of the total 455 patients, CD4^+^ T-cell counts were available for 227 individuals, while 228 patients had no CD4^+^ data. Among those with available CD4^+^ counts:

- 16 of 227 patients had CD4^+^ counts <200/μL. Among them, 9 were HIV-negative and had liver cirrhosis, forming IRC A (n = 9). The remaining 7 patients were HIV-positive with asymptomatic non-alcoholic fatty liver disease (NAFLD, now termed metabolic dysfunction–associated steatotic liver disease: MASLD) and were assigned to IRC B (n = 7).
- 211 of 227 patients had CD4^+^ counts ≥200/μL. Of these, 205 patients had CD4^+^ counts ≥500/μL and were not included in any cluster. The remaining 6 patients had intermediate CD4^+^ counts (200–499/μL), were HIV-negative, and all 6 had liver cirrhosis, forming IRC C (n = 6).

### 3.3. HIV Status and Disease Stage

Distribution of selected characteristics among HIV-positive patients in IRC B—including classification of HIV-Infection—was based on the 2014 revised Centers for Disease Control and Prevention (CDC) clinical staging system, as presented in Table 2.

- CDC-A: Asymptomatic HIV infection
- CDC-B: Symptomatic conditions not meeting the AIDS-defining criteria
- CDC-C: AIDS-defining conditions

This classification depicts the clinical spectrum of HIV disease and guides both surveillance and treatment decisions.

Two patients (CDC stage C) presented with high-level HIV-RNA viremia (10^4^–10^5^ copies/mL) and AIDS-defining illnesses in conjunction with severe coinfections (Supplementary Information S1: Table S3a). Five patients (CDC stage B) had detectable HIV-RNA in the range of 10^2^–10^5^ copies/mL (Supplementary Information S1: Table S3b); all 7 patients initiated antiretroviral therapy (ART) within 10 days of hospital admission.

### 3.4. Liver Disease Severity and Prognosis

Liver disease severity and IHM in cirrhotic patients were evaluated using the Child-Pugh Score (CPS) [23]. Patients without cirrhosis—including seven HIV-positive individuals with MASLD (classified as IRC B) and three post–LTx cases from IRC A that lacked clinical or biochemical evidence of liver dysfunction—were excluded from CPS-based analyses; here, CPS entries were marked as not applicable (N/A). Thus, subsequent results refer exclusively to the subset of 12 patients with confirmed cirrhosis (Supplementary Information S1: Table S5).

Table 3 shows that IHM increased consistently with the LHC and CPS stage. A single patient managed exclusively on a RCW survived and had a CPS of 5 points (CPS stage A). In contrast, IHM rose to 71.4% among those admitted to RCW and/or ImCU, and to 75.0% in patients requiring both ImCU and ICU support. Within these higher-acuity groups, CPS ranged predominantly within stages B (7–9 points) and C (10–15 points), with corresponding increases in observed IHM.

To contextualize the observed mortality in relation to CPS-based expectations, Table 3 summarizes median CPS scores, stages, and predicted 1- and 2-year mortality risk among survivors and non-survivors in IRC A and C. Among IRC C patients, non-survivors had a median CPS of 9 points (stage B), exceeding the score of survivors (7 points). In IRC A, the discrepancy was even more pronounced: non-survivors had a median CPS of 12 points (stage C), compared to 9 points in survivors. These scores correspond to estimated 1-year mortality rates of 30–40% for stage B and 50–60% for stage C [23].

### 3.5. CD4^+^ T-Cell Counts and Immune Profiles

Distribution of available CD4^+^ T-cell count measurements across all LHC, i.e., RCW, ImCU and ICU, and across all IRCs are provided in Table 4.

Among 227 (49.9%) of 455 hospitalized patients, CD4^+^ T-cell counts were available for 217 of 352 survivors (61.6%) and 10 of 103 non-survivors (9.7%), corresponding to 47.7% and 2.2% of the total cohort (n = 455), respectively.

CD4^+^ T-cell counts were available for all 22 patients assigned to IRC A (n = 9), IRC B (n = 7), and IRC C (n = 6). This includes the single patient with CPS stage A (CD4^+^ count: 471/μL), who belonged to the 206 RCW-only survivors.

The vast majority of CD4^+^ measurements (206/227; 90.7%) were obtained from survivors admitted to RCW only. A smaller fraction originated from patients requiring higher levels of care: 15/227 (6.7%) from the ImCU and 6/227 (2.6%) from the ICU.

IRC A showed the lowest median CD4^+^/μL per IRC, i.e., A vs. B vs. C: 102/μL vs. 171/μL vs. 263/μL, and per IHM outcome (within each IRC), i.e., non-survivors vs. survivors: 56.3/μL vs. 119/μL (A) or 162.5/μL vs. 183/μL (B) or 281.5/μL vs. 363/μL (C). Of note, the median CD4^+^ T-cell count among the 206 RCW-only survivors was 683/μL, reflecting substantially preserved immune status in this subgroup compared to patients admitted to higher LHC.

These findings emphasize a strong association between CD4^+^ T-cell depletion, higher care needs, and IHM, underscoring the clinical value of early immune assessment—particularly in patients with liver disease, advanced immunosuppression, or both.

In IRC-A (CD4^+^ < 200/μL), PJ PCR positivity reached 37.5%, with no PJP-related deaths; IHM was 55.6%, driven by complications of end-stage liver disease. IRC-B (HIVpositive) showed 28.6% mortality, including one PCR-positive survivor with PJP requiring therapy with trimethoprim–sulfamethoxazole (TMP–SMX). IRC-C (CD4^+^ 200–499/μL) had an IHM of 50%, and its sole PJ PCR-positive patient died. All ICU-admitted IRC-B (CDC stage C) and IRC-C patients died (Supplementary Information S1: Tables S3a and S4a).

### 3.6. Fungal Burden: Pneumocystis jirovecii PCR and _β_-D-glucan (BDG) Biomarker Detection

A total of 8 patients were tested for PJ by PCR (IRC-A: 2; IRC-B: 4; IRC-C: 2) out of 22 individuals with CD4 200–499/μL (IRC-C) and CD4 < 200/μL (IRC-A and IRC-B). The overall PCR positivity rate was 37.5%, with 3 of the 8 tested patients yielding PJ DNA. When calculated across all IRCs, PCR positivity corresponded to 11.1% in IRC A (1/9), 14.3% in IRC B (1/7), and 16.7% in IRC C (1/6). The cycle threshold (Ct) values among PCR-positive patients ranged from 27 to 31.7, consistent with intermediate to low fungal burden (Table 5). Notably, two cirrhotic patients (one each in IRC-A and IRC-C)—both with abnormal chest imaging—survived without specific treatment for PJP, potentially attributable to their relatively low PJ load suggested by intermediate Ct values. The third PCR-positive case was an HIV-positive individual with MASLD and a CD4^+^ T-cell count of 183/μL who also had abnormal chest imaging and received TMP–SMX treatment without BDG testing.

Regarding serum BDG testing, no results were available for the three PJ PCR-positive patients, which limited our ability to correlate BDG levels with fungal burden or clinical outcomes. The lack of BDG testing in these cases reflects clinical decision-making including treatment strategies guided by detection of other pneumonia causing pathogens and the frequent presence of significant multisite *Candida* colonization in cirrhotic patients (Supplementary Information S1: Tables S2a,b, S3a,b and S4a,b).

The analysis also covered B cells (CD19^+^), cytotoxic CD8^+^ T cells, NK cells (CD3^−^CD16/56^+^), and activated T cells (CD3^+^HLA-DR^+^) to contextualize immune competence across subgroups (see Supplementary Information S1: Table S1).

## 4. Discussion

This study investigated the relationship between peripheral CD4^+^ (and CD8^+^) T-cell counts and OI, particularly PJP, in hospitalized patients with liver disease, focusing on HIV-negative cirrhotic patients and including HIV-positive MASLD patients as a separate comparator group. This study was not designed to estimate incidence of PJP in cirrhosis; rather, it provides descriptive, hypothesis-generating insights into CD4^+^ depletion patterns and their association with PJ detection. By stratifying patients according to CD4^+^ counts, we assessed the impact of T-cell depletion on PJ-related morbidity and mortality—a topic previously underexplored in the context of cirrhosis. The central finding is that profound CD4^+^ T-cell depletion, while common in cirrhosis and associated with increased PJ detection, did not translate into PJ-driven mortality.

Severe CD4^+^ T-cell depletion (<200/μL) was linked to higher likelihood of PJ detection (50% PCR-positivity rate among tested cirrhotic patients, although n = 2/4) but not consistently to higher mortality. Among the three PJ PCR-positive patients, only one (IRC-C, Ct 28.9) died; two survived—one IRC-A cirrhotic (Ct 27) without specific prophylaxis/therapy and one HIV-positive patient with MASLD (IRC-B, Ct 31.7) under TMP–SMX treatment. These outcomes suggest two key points. First, CD4^+^ depletion increases the likelihood of PJ detection in cirrhosis, confirming that advanced immune dysfunction predisposes to colonization or low-grade infection. Second, fungal burden and clinical context appear to drive outcomes more strongly than PJ PCR Ct thresholds alone. The survival of a Child–Pugh B cirrhotic with severe CD4^+^ depletion (102/μL) and PCR-confirmed PJ-Detection (IRC-A, Ct 27) without specific therapy supports the idea that moderate fungal loads may be effectively controlled even in advanced liver disease when residual immunity persists. Reduced CD4^+^ T-cell counts in cirrhosis likely reflect immune-paralysis in advanced disease, driven by systemic inflammation and bacterial translocation. This immune dysfunction predisposes patients to opportunistic infections and highlights that lymphopenia can be a marker of broader immunologic compromise rather than an isolated finding [12,13].

In this study, All-cause in-hospital mortality among PJ PCR-positive patients was 33.3% (1/3), with no death adjudicated as PJP-attributable. Among cirrhotic patients with CD4^+^ < 500/μL mortality reached 50% (noting the very small sample size: n = 1/2). This aligns with non-HIV PJP mortality rates of ~30% reported in meta-analyses (which did not include liver diseases) [25], but it is markedly lower than previously reported in cirrhotic populations (see comparison with earlier studies) [26,27]. Importantly, high in-hospital mortality across IRCs (IRC-A: 55.6%; IRC-C: 50%) was driven by hepatic decompensation and bacterial infections, not by opportunistic fungal disease [28] (see Supplementary Information S1: Comment for Table S1). Even profoundly immunosuppressed cirrhotic patients with CD4^+^ < 200/μL did not develop PJP despite the absence of specific prophylaxis. This contrasts with Mansharamani et al., who found CD4^+^ < 300/μL in 91% of non-HIV PJP cases and proposed using CD4^+^ monitoring to guide chemoprophylaxis; however, their study effectively excluded liver cirrhosis [29].

As already stated, previous studies described higher mortality associated with PJP in cirrhosis. Franceschini et al. reported eight decompensated cirrhotics with PCR-confirmed PJP, five of whom (62.5%) died within 30 days [26]. Peschel et al. reported 60% overall mortality and 84.7% mortality among Child-Pugh C patients in a cohort of 151 ICU cases with PJP (67 with cirrhosis), identifying cirrhosis as an independent 4.8-fold risk factor for death [27]. Notably, neither study incorporated lymphocyte phenotyping or stratified outcomes by CD4^+^ or CD8^+^ counts [26,27], leaving the underlying degree of immune dysfunction potentially more heterogeneous than described. In our cohort, Child-Pugh C cirrhotics exhibited a similarly high ICU mortality of 83.4%. Again, in contrast to non-HIV immunosuppressed cohorts where CD4^+^ <300/μL is strongly associated with adverse outcomes [29], our data indicate that peripheral CD4^+^ lymphopenia primarily increases susceptibility to PJ rather than conferring PJP-specific mortality. This aligns with Aguilar et al. and Damiani et al., who demonstrated that many PCR-based detections in immunocompromised patients—particularly those with intermediate Ct values—reflect colonization rather than true PJP unless supported by adjunct markers such as serum β-D-glucan [30,31]. Given its diagnostic value in borderline cases, BDG could have served as a reliable discriminator in our cohort; however, its non-systematic assessment and the potential for confounding by *Candida* spp. multi-site colonization limited its interpretability. In our study, fatal outcomes aligned with hepatic decompensation, organ failure, and superimposed bacterial infections—hallmarks of CAID [12,14,28].

These findings have implications for risk stratification, diagnostic interpretation, and prophylaxis in cirrhosis. While TMP–SMX prophylaxis is standard in HIV/AIDS patients with CD4^+^ < 200/μL [3–5], hepatology guidelines (by EASL or the American Association for the Study of Liver Diseases, AASLD or the British Society of Gastroenterology, BSG) lack corresponding advice except in autoimmune hepatitis [32–34]. Our data suggest that a subset of cirrhotics with CD4^+^ < 200/μL exhibit immunological profiles resembling advanced immunodeficiency, yet their mortality is driven by hepatic rather than opportunistic disease. Still, several researchers have long proposed using CD4^+^ counts to guide PJP prevention. Mansharamani et al. and Avino et al. advocated CD4^+^ < 300/μL and CD4^+^ < 200/μL, respectively, in non-HIV populations [29,35]. Peschel et al. similarly conclude that PJP prophylaxis should be “considered at an early stage” in cirrhosis [27]. Franceschini et al. also recommend PJP prophylaxis for decompensated cirrhotics receiving steroids [26].

The role of PJP prophylaxis in cirrhosis is therefore nuanced, and recommendations remain inconsistent across specialties [32–34]. Importantly, PJP incidence even post-LTx or in ANCA-vasculitis patients on rituximab is low (~1.2%), and routine prophylaxis is not uniformly endorsed [18,36,37]. Nevertheless, Nature Reviews Gastroenterology & Hepatology identified advanced cirrhosis and concomitant immunosuppression as PJP risk factors and suggested targeted prophylaxis [12]. Reported colonization rates in advanced liver disease remain rare (1.4%) [17], but it remains unclear whether these reflect systematic screening of all admissions (e.g., routine sputum sampling) or only selective, clinically triggered testing—limiting comparability and interpretability. Given this uncertainty, PJ detection in cirrhosis may also carry infection-control relevance. Recent studies demonstrated possible nosocomial transmission, including airborne spread from colonized patients in transplant units [38,39]. Because cirrhotic patients often share clinical environments with LTx recipients, understanding PJ colonization dynamics could support preventive strategies even if clinical PJP remains infrequent.

Given our results and the high mortality observed—in line with previous reports for patients with decompensated cirrhosis [40,41]—, we propose that CD4^+^ counts be incorporated into cirrhosis risk stratification. Cirrhotics with CD4^+^ < 200/μL may benefit from early diagnosis of severe CAID and from targeted PJP prevention and testing (via PCR with cycle counts along with BDG), particularly when additional risk factors such as drug-induced immunosuppression (e.g., post-LTx) or exposure to PJ-colonized/-infected index patients are present, paralleling established HIV practice [5]. Prospective trials are needed to determine whether such interventions improve survival. Until then, given the limited number of PJ-PCR–positive cases and the strong impact of cirrhosis-related complications and bacterial infections on overall mortality, our findings should be regarded as hypothesis-generating rather than definitive.

This study has several limitations as the retrospective single-center design and the small sample size of the IRCs limit the generalizability of our findings. First, BDG testing was not available for the three PJ PCR-positive patients (see Supplementary Information S1: Tables S2a,b, S3a,b and S4a,b). Second, the sample size of cirrhotic patients with available CD4^+^ counts—particularly those tested for PJ by PCR—was small, limiting statistical inference and generalizability. PJ PCR testing was performed only in patients with abnormal radiological chest imaging or as part of pre-LTx evaluations, which may introduce diagnostic bias and could underestimate the true prevalence of PJ in the cohort. Third, CD4^+^ T-cell counts were available almost exclusively in survivors (61.6%) but rarely in non-survivors (9.7%), leading to substantial selection bias. Therefore, associations between CD4^+^ depletion and mortality in the cirrhotic population must be interpreted with extreme caution and that our findings are hypothesis-generating rather than definitive. Fourth, immune phenotyping beyond CD4^+^ counts (e.g., CD8^+^, NK cells, activated T cells) was available only for a subset of patients, and many data were derived from clinically triggered rather than systematic testing. Given the very small effective sample size, particularly for PJ PCR-positive patients, we refrained from multivariable modelling. All analyses are therefore descriptive and unadjusted, and residual confounding by disease severity, steroid use, other immunosuppressants, or comorbidities is likely. Additionally, for HIV-positive patients, ART follow-up, viral suppression timing, and immune reconstitution data were unavailable, limiting longitudinal analysis. Finally, the retrospective single-center design introduces potential selection bias and limits the ability to establish causal relationships.

## 5. Conclusions

Cirrhosis is a clinically relevant immunocompromised state, with a broad spectrum of immunodysregulation ranging from hyper-inflammatory states to immune exhaustion and paralysis. Patients with cirrhosis are vulnerable to a wide array of infections—bacterial, fungal, and, less commonly, opportunistic—that markedly increase short-term mortality [28]. In this study, severe peripheral CD4^+^ T-cell depletion was common in cirrhosis and associated with PJ detection; however, PJP-specific mortality remained low. Overall outcomes were primarily driven by hepatic decompensation and bacterial infections rather than PJP. While our findings suggest that CD4^+^ assessment may help inform risk stratification, these results are exploratory and hypothesis-generating. Further prospective studies are required to determine whether CD4-guided strategies for opportunistic infection prevention, diagnostic evaluation, or targeted PJP prophylaxis are clinically effective and safe in this population.

## Supplementary Material

supplemental 1

supplemental 2

**Supplementary Materials:** The following supporting information can be downloaded at: https://www.mdpi.com/article/10.3390/livers6030040/s1, Supplementary Information S1: Table S1. Immunological cell subsets and infection-related clinical outcomes across IRCs; Table S2a: IRC A died (n = 5); Table S2b: IRC A survived (n = 4); Table S3a: IRC B died (n = 2); Table S3b: IRC B survived (n = 5); Table S4a: IRC C died (n = 3); Table S4b: IRC C survived (n = 3); Table S5. Demographic characteristics, clinical and laboratory parameters, and Mortality Risk Stratification at admission to hospital of patients with liver disease (n = 15) and CD4^+^ < 200/μL or 200–499/μL assigned to IRC A (n = 9) or IRC C (n = 6), respectively. Of those, 3 were post Liver Transplantation and 12 with active liver cirrhosis. Supplementary Information S2 [31,42].

## Figures and Tables

**Table 1.** LHC and corresponding IHM rates across the full study cohort and within each IRC.

Patients Requiring Admission to	n	In-Hospital Mortality (%)	IRC per LHC					
			IRC-A		IRC-B		IRC-C	
			(n = 9)		(n = 7)		(n = 6)	
			Died	Survived	Died	Survived	Died	Survived
RCW only	285	6 (2.1%)	-	-	-	-	-	1
RCW and/or ImCU	121	51 (42.1%)	4	3	-	5	1	2
RCW and/or ImCU and ICU	49	46 (93.8%)	1	1	2	-	2	-
Total	455	103 (22.6%)	5	4	2	5	3	3
IHM %		22.6%	55.6%		28.6%		50.0%	

LHC: Level of required Hospital Care. IHM: In-Hospital Mortality. IRC: Immunological Risk Cluster. RCW: Regular Care Ward. ImCU: Intermediate Care Unit. ICU: Intensive Care Unit.

**Table 2.** Distribution of HIV-patients in IRC B by LHC and CDC classification of HIV infection.

IRC B: HIV-Patients Requiring Admission to	n	In-Hospital Mortality (%)	CDC Classification * of HIV Infection per LHC					
			CDC-A		CDC-B		CDC-C	
			(N/A)		(n = 5)		(n = 2)	
			Died	Survived	Died	Survived	Died	Survived
RCW only	-	-	-	-	-	-	-	-
RCW and/or ImCU	5	0 (0.0%)	-	-	-	5	-	-
RCW and/or ImCU and ICU	2	2 (100.0%)	-	-	-	-	2	-
Total	7	2 (28.6%)	-	-	-	5	2	-
IHM %		28.6%	-		0.0%		100.0%	

HIV: Human Immunodeficiency Virus. IRC: Immunological Risk Cluster. LHC: Level of required Hospital Care. CDC: Center for Disease Control and Prevention. RCW: Regular Care Ward. ImCU: Intermediate Care Unit. ICU: Intensive Care Unit. IHM: In-Hospital Mortality.

* Revised surveillance case definition for HIV infection–United States, 2014. MMWR Recomm Rep, 2014. 63(Rr-03): pp. 1–10 [22].

**Table 3.** CPS (points, median), expected and observed IHM in cirrhotic patients (n = 12) stratified by LHC and IRC in two clusters (IRC A and IRC C), excluding three post-LTx cases ^‡^ and seven IRC B cases ^†^ with HIV.

Patients Requiring Admission to	n	In-Hospital Mortality (%)	Child-Pugh Score ** (CPS: Points, Median) per IRC and LHC					
			IRC-A		IRC-B		IRC-C	
			(n = 6)		(n = 0)		(n = 6)	
			Died	Survived	Died	Survived	Died	Survived
RCW only	1	0/1 (0.0%)	-	-	-	-	-	5
RCW and/or ImCU	7	5/7 (71.4%)	11	N/A ^‡^	-	N/A ^†^	10	7
RCW and/or ImCU and ICU	4	3/4 (75.0%)	13	9	N/A ^†^	-	9	-
Total	12	8/12 (66.7%)	12	9	N/A ^†^	N/A ^†^	9	7
IHM %		66.7%	83.4%		N/A ^†^		50.0%	
Expected 1-Year Mortality based on CPS			~50–60%				~30–40%	
Expected 2-Year Mortality based on CPS			~70–80%				~40–60%	

CPS: Child-Pugh Score. IHM: In-Hospital Mortality. LHC: Level of required Hospital Care. IRC: Immunological Risk Cluster. HIV: Human Immunodeficiency Virus. LTx: Liver Transplantation. RCW: Regular Care Ward. ImCU: Intermediate Care Unit. ICU: Intensive Care Unit. IHM in this cohort exceeded expected prognostic estimates, particularly in IRC A. These findings suggest that although CPS remains useful for stratifying hepatic risk at admission, it may underestimate acute mortality in immunocompromised cirrhotic patients requiring high-level care. The divergence between observed and Simplified Acute Physiology Score 3 (SAPS 3)–predicted mortality (Supplementary Information S1: Table S5) further indicates that severe CD4^+^ T-cell depletion—classically linked to HIV—can also occur in cirrhosis, contributing to adverse short-term outcomes (Supplementary Information S1: Table S1).

** Child, C.G. and J.G. Turcotte, Surgery and portal hypertension. Major Probl Clin Surg, 1964. 1: pp. 1–85 [24].

**Table 4.** Distribution of peripheral CD4^+^ T-cell counts in hospitalized patients stratified by LHC, IHM, and IRCs.

Patients Requiring Admission to	n	Patients with CD4+ Count		CD4^+^ Count (Cells/μL, Median) per IRC and LHC					
				IRC-A		IRC-B		IRC-C	
				(n = 9)		(n = 7)		(n = 6)	
		Died	Survived	Died	Survived	Died	Survived	Died	Survived
RCW only	285	0/6	206^¥^/279	-	-	-	-	-	471^◊^
RCW and/or ImCU	121	5/51	10/70	71.5	136	-	183	253	255
RCW and/or ImCU and ICU	49	5/46	1/3	41	102	162.5	-	310	-
n	455 *	10/103	217/352						
%		9.7% * 2.2%	61.6% * 47.7%						
median CD4^+^ /μL per IHM Outcome				56.3	119	162.5	183	281.5	363
median CD4^+^ /μL per IRC				102		171		263	

* The values 2.2% and 47.7% correspond to the proportions of non-survivors (10/455) and survivors (217/455), respectively, among all 455 patients with available CD4^+^ counts.

¥ Median CD4^+^ count for 206 patients was 683/μL.

◊ All 206 RCW-only survivors subjected to this analysis had available CD4^+^ values, including the CPS stage A patient (471/μL) in IRC C. LHC: Level of required Hospital Care. IHM: In-Hospital Mortality. IRC: Immunological Risk Cluster. RCW: Regular Care Ward. ImCU: Intermediate Care Unit. ICU: Intensive Care Unit.

**Table 5.** Detection of *Pneumocystis jirovecii* (PJ) detection (via PCR) among patients stratified by IRC, LHC, and IHM (n = 22).

Patients Requiring Admission to	n	*Pneumocystis jirovecii* PCR +					
		IRC-A		IRC-B		IRC-C	
		(n = 9)		(n = 7)		(n = 6)	
		Died	Survived	Died	Survived	Died	Survived
RCW only	1	-	-	-	-	-	0/1
RCW and/or ImCU	15	0/4	0/3	-	1/5	0/1	0/2
RCW and/or ImCU and ICU	6	0/1	1/1	0/2	-	1/2	-
Total	22	5	4	2	5	3	3
Not tested (N/A)	14	5	2	0	3	1	3
Tested negative	5	0	1	2	1	1	0
Tested positive	3	0	1	0	1	1	0
Ct Value (PCR)	-	-	28.27	-	31.70	28.92	-
PJ PCR-positivity rate among tested patients	3/8 37.5%	1/2 50.0%		1/4 25.0%		1/2 50.0%	
PJ PCR-positivity rate among all patients	3/22 13.6%	1/9 11.1%		1/7 14.3%		1/6 16.7%	

LHC: Level of required Hospital Care. IHM: In-Hospital Mortality. IRC: Immunological Risk Cluster. RCW: Regular Care Ward. ImCU: Intermediate Care Unit. ICU: Intensive Care Unit.

## Data Availability

The data supporting the findings of this study are available from the corresponding author upon reasonable request.

## Abbreviations

aPTT: activated partial thromboplastin time
BAL: Bronchoalveolar Lavage
CCI: Charlson Comorbidity Index
CMV: Cytomegalovirus
CRP C: Reactive Protein
LHC: Level of required Hospital Care (i.e., RCW, ImCU, ICU)
HSV: Herpes Simplex Virus
ICU: Intensive Care Unit
ImCU: Intermediate Care Unit
INR: International Normalized Ratio
IRC: Immunological risk cluster
MD: Median
N/A: Not applicable/Not available
PJ, PJP: PPneumocystis jirovecii Pneumocystis jirovecii pneumonia
PRW: Pharyngeal Rinse Water
Q1: First quartile (25th percentile)
Q3: Third quartile (75th percentile)
RCW: Regular Care Ward
Rx: Radiological imaging (thorax: CT, X-ray) showing atypical or mixed pulmonary infiltrates
SAPS: Simplified Acute Physiology Score
TG: Toxoplasma gondii
VRE: Vancomycin resistant *Enterococcus faecium*
