# Supplementary material for: Are CD4+ T-Cell Counts Associated with Pneumocystis jirovecii Detection in Hospitalized Patients with Liver Disease? A Retrospective Exploratory Pilot Analysis
Source: Livers. Author manuscript; Available in PMC 2026 Jun 2. (PMC13225858; doi:10.3390/livers6030040)
Supplement: supplemental 1 [file NIHMS2179768-supplement-supplemental_1.pdf]

Supplementary Information 1

Table S1. Immunological cell subsets and infection-related clinical outcomes across IRCs.

Variable	IRC A (n = 9)	IRC B (n = 7)	IRC C (n = 6)	Total (n = 22)	p-value
**In-hospital mortality**	5 (55.6)	2 (28.6)	3 (50.0)	10 (45.5)	0.5681
**ICU admission**	2 (22.2)	2 (28.6)	2 (33.3)	6 (27.3)	>0.9999
***Pneumocystis jirovecii* PCR positivity**	1 (11.1)	1 (14.3)	1 (16.7)	3 (13.6)	>0.9999
**Toxoplasma gondii IgG seropositivity**	4 (44.4)	1 (14.3)	2 (33.3)	7 (31.8)	0.4488
**Detection of any bacteria in blood culture**	7 (77.8)	5 (71.4)	2 (33.3)	14 (63.6)	0.2069
**Detection of Gram-positive bacteria in blood culture**	6 (66.7)	3 (42.9)	2 (33.3)	11 (50.0)	0.5626
**Detection of Gram-negative bacteria in blood culture**	1 (11.1)	2 (28.6)	0 (0.0)	3 (13.6)	0.4497
**T CD3⁺ (%)**	81.4 (59.4–86.3)	80.7 (74.4–86.9)	74.7 (68.1–81.3)	80.5 (68.5–85.3)	0.3215
**T CD3⁺ (/µl)**	206 (93–298)	1500 (899–2290)	435 (373.5–520.5)	435 (218–900.3)	**0.0009**
**B CD19⁺ (%)**	6.2 (4.3–21.8)	7.9 (4.7–14.8)	15.5 (6.1–22.8)	8.3 (5.0–17.9)	0.6721
**B CD19⁺ (/µl)**	25 (10.5–38)	117 (71–382)	96 (39–133.3)	62.5 (24–117.3)	**0.0180**
**Th CD3⁺/CD4⁺ (%)**	36.6 (25.9–40.2)	51.9 (44.8–64.4)	48.8 (42.5–57.8)	44.3 (34.6–52.1)	**0.0239**
**Th CD3⁺/CD4⁺ (/µl)**	102 (37.5–141.5)	171 (102–188)	263 (246.8–395.3)	162.5 (97.5–240.3)	**0.0013**
**Tc CD3⁺/CD8⁺ (%)**	42.6 (27.0–51.1)	24.3 (16.3–36.3)	20.1 (17.5–29.6)	28.1 (19.7–43.9)	0.0961
**Tc CD3⁺/CD8⁺ (/µl)**	84 (53–153.5)	400 (286–616)	139 (97.3–150.5)	141 (85.5–331)	**0.0054**
**NK CD3⁻, CD16/56⁺ (%)**	8.6 (7.5–16.3)	7.8 (7.1–14.7)	9.1 (7.1–13.6)	8.7 (7.3–13.1)	0.5951
**NK CD3⁻, CD16/56⁺ (/µl)**	21 (17–48.5)	168 (78–281)	57.5 (40.8–84.5)	58.5 (22.5–98)	**0.0050**
**Tcyt CD3⁺, CD16/56⁺ (%)**	6.7 (3.9–11.0)	3.5 (1.5–5.1)	2.4 (1.5–4.1)	3.8 (2.1–6.9)	**0.0085**
**CD3⁺, HLA-DR⁺ (%)**	22.2 (14.4–44.2)	28.5 (8.0–33.0)	16.3 (5.5–19.3)	18.5 (11.1–30.2)	0.1706
**CD3⁺, HLA-DR⁺ (/µl)**	38 (26.5–134.5)	293 (209–560)	94 (34.3–128)	118 (37–266.8)	**0.0086**
**Ratio CD3/CD8 (%)**	0.9 (0.6–1.4)	2.0 (1.4–4.0)	2.2 (1.6–3.3)	1.7 (0.9–2.3)	**0.0353**

Table S1. *Immunological risk cluster (IRC) A = HIV-negative cirrhosis with CD4⁺ < 200/µl; IRC B = HIV-positive patients with CD4⁺ < 200/µl and non-cirrhotic NAFLD; IRC C = HIV-negative cirrhosis with CD4⁺ 200–499/µl; Th = T-helper (CD3⁺/CD4⁺);*

Supplementary Information 1

Tc = cytotoxic T-cells ($CD3^+/CD8^+$); NK = natural killer cells ($CD3^-$, $CD16/56^+$); Tcyt = $CD3^+$, $CD16/56^+$ NK-like cytotoxic T-cells ; HLA-DR = human leukocyte antigen – DR isotype; NAFLD = non-alcoholic fatty liver disease; MASLD = metabolic dysfunction-associated steatotic liver disease. Data are expressed as median (Q1–Q3) or n (percentage), as appropriate. p-values were obtained using the Kruskal–Wallis test for continuous variables and the Fisher exact test for categorical variables. Significant p-values (< 0.05) are indicated in bold.

Comment for Table S1 on Associations Between Immunocyte Subsets and Clinical Outcomes in Immunological Risk Clusters (IRCs):

Table S1 summarizes immunological cell subset distributions and infection-related outcomes across three IRCs (A–C) in patients with liver disease.

In **IRC B** (HIV-positive patients with $CD4^+ < 200/\mu l$ and non-cirrhotic NAFLD, now termed MASLD, IRC B), a decline in NK $CD3^-$, $CD16/56^+$ cells (%) was associated with higher in-hospital mortality (OR 0.45, 95% CI 0.006–1.00, p = 0.051) and an increased likelihood of ICU admission (OR 0.45, 95% CI 0.006–1.00, p = 0.051), narrowly missing statistical significance. Detection of Gram-positive bacteria in blood cultures was significantly associated with lower absolute NK $CD3^-$, $CD16/56^+$ cell counts (OR 0.97, 95% CI 0.89–0.99, p = 0.023), reduced cytotoxic T-cell ($CD3^+$, $CD8^+/\mu l$) counts (OR 0.99, 95% CI 0.96–1.00, p = 0.033), and an increased CD4/CD8 ratio (OR 10.93, 95% CI 1.18–185.2, p = 0.027).

These findings suggest that depletion of NK and $CD8^+$ T-cells, together with an elevated CD4/CD8 ratio, may contribute to impaired antibacterial immune responses and higher infection-related morbidity among immunocompromised HIV-positive patients with non-cirrhotic NAFLD (now termed MASLD).

When combining **IRC A** (HIV-negative patients with liver cirrhosis and $CD4^+ < 200/\mu l$) **and C** (HIV-negative patients with liver cirrhosis and $CD4^+$ 200–499/µl), the detection of any bacteria in blood culture was significantly associated with lower proportions of Th $CD3^+/CD4^+$ T-cells (OR 0.89, 95% CI 0.74–0.99, p = 0.028), higher proportions of Tc $CD3^+/CD8^+$ T-cells (OR 1.12, 95% CI 1.01–1.35, p = 0.022), and a markedly reduced CD4/CD8 ratio (OR 0.21, 95% CI 0.02–0.86, p = 0.023).

Similarly, detection of Gram-positive bacteria in blood culture showed consistent trends, with lower Th $CD3^+/CD4^+$ T-cell frequencies (OR 0.88, 95% CI 0.74–0.99, p = 0.022), higher Tc $CD3^+/CD8^+$ T-cell frequencies (OR 1.07, 95% CI 0.99–1.21, p = 0.077), and a lower CD4/CD8 ratio (OR 0.30, 95% CI 0.04–1.02, p = 0.055), narrowly missing statistical significance.

These findings suggest that in immunocompromised HIV-negative patients with liver cirrhosis, bacterial bloodstream infection is associated with a shift toward cytotoxic immune dominance, characterized by relative $CD4^+$ T-cell depletion and a reduced CD4/CD8 ratio.

When all immunological risk clusters were analyzed together, ICU admission was significantly associated with increased mortality in these immunocompromised patients with liver disease (OR 11.0, 95% CI 1.33–242.6, p = 0.0248). Detection of Gram-positive bacteria in blood cultures was also associated with a markedly higher likelihood of ICU admission (OR 8.33, 95% CI 1.02–181.3, p = 0.0477) and showed a trend toward a 4.6-fold increase in-hospital mortality (OR 4.67, 95% CI 0.82–32.8, p = 0.083).

Among patients tested for *Pneumocystis jirovecii* (PJ) by PCR in bronchoalveolar lavage (BAL), PJ-PCR positivity was associated with a lower median proportion of Th $CD3^+/CD4^+$ T-cells (43.7% vs. 54.0%, p = 0.114) and a higher median proportion of Tc $CD3^+/CD8^+$ T-cells (36.3% vs. 20.3%, p = 0.057). Consequently, the CD4/CD8 ratio was reduced in PJ-PCR–positive patients (median 1.4 vs. 2.4, p = 0.057), although these differences did not reach statistical significance.

Supplementary Information 1

Table S2a and S2b (IRC A). Required hospital care level (LHC) and in-hospital mortality (IHM) rates for participants within Immunological risk cluster A (IRC A).

Table S2a: IRC A died (n=5)

	CD4+ count/µL	R_xCI	N-ß-D-glucan (serum)	Culture (BAL, Sputum, Tracheal Fluid)	PCR (BAL) PJ	PCR (BAL) CMV	PCR (BAL) HSV	Culture (blood, faeces, urine, ascites, swab)	TG IgG (AU/mL)
RCW and/or ImCU									
P2	30	abn	274	*N/A*	*N/A*			*S. epidermidis* (a)<br>*E. faecium* (a)<br>*C. glabrata* (a)<br>*Lactobacillus paracasei* (b)	+<br>(24)
P6	147	abn	288	*N/A*	*N/A*			*C. albicans* (u)	-
P5	109	n	*N/A*	*N/A*	*N/A*			*S. epidermidis* (b)	-
P9	34	abn	*N/A*	*N/A*	*N/A*			*S. aureus* (b)<br>*S. aureus* (a)	+<br>(50)
RCW and/or ImCU and ICU									
P4	41	n	*N/A*	*Aspergillus fumigatus* (Sp)	*N/A*			*S. aureus/MRSA* (s)<br>*S. aureus/MRSA* (a)<br>*S. epidermidis* (b)<br>*C. albicans* (f)<br>*C. glabrata* (f)	-

Table S2b: IRC A survived (n=4)

	CD4+ count/µL	R_xCI	N-ß-D-glucan (serum)	Culture (BAL, Sputum, Tracheal Fluid)	PCR (BAL, PRW) PJ	PCR (BAL, PRW) CMV	PCR (BAL, PRW) HSV	Culture (blood, faeces, urine, ascites, swab)	TG IgG (AU/mL)
RCW and/or ImCU									
P1 ‡	84	abn	*N/A*	*C. albicans* (Sp)	-<br>(PRW)	+<br>(PRW)	+<br>(PRW)	*S. aureus* (b)<br>*K. oxytoca* (b)<br>*K. pneumoniae* (u)<br>*P. aeruginosa* (u)<br>*Cl. difficile* (f)	+<br>(5)
P3 ‡	173	n	*N/A*	*N/A*	*N/A*			E. coli b	-
P8 ‡	136	abn	*N/A*	*N/A*	*N/A*			*K. pneumoniae* (b)<br>*E. faecium* (b)<br>*C. albicans* (u)<br>*C. glabrata* (u)<br>*E. faecium/VRE* (s)	+<br>(45)
RCW and/or ImCU and ICU									
P7	102	abn	*N/A*	*E. faecalis* *(BAL, TF)*<br>*C. albicans (TF)*<br>*E. coli (TF)*	+<br>(BAL)<br>Ct: 28,27	+<br>(BAL)	+<br>(BAL)	*E. faecalis* (b)<br>*S. epidermidis* (b)<br>*Actinomyces naeslundii* (b)	-

Table S2a and S2b (IRC A). *Levels of care: RCW (Regular Care Ward), ImCU (Intermediate Care Unit), ICU (Intensive Care Unit). Radiological chest imaging (R_xCI): n = normal; abn = abnormal. Sample sources: a = ascites; b = blood; Sp = sputum; s = swab; u = urine; f = faeces; TF = tracheal fluid. BAL: bronchoalveolar lavage. PRW: pharyngeal rinse water. PJ: Pneumocystis jirovecii. CMV: cytomegalovirus; HSV: herpes simplex virus. TG: Toxoplasma gondii. N/A: not applicable. Post Liver Transplantation ‡.*

Supplementary Information 1

Table S3a and S3b (IRC B). Required hospital care level (LHC) and in-hospital mortality (IHM) rates for participants within Immunological risk cluster B (IRC B).

Table S3a: IRC B died (n=2)

RCW and/or ImCU and ICU									
	CD4+ count/µL	R_xCI	N-ß-D-glucan (serum)	Culture (BAL, Sputum, Tracheal Fluid)	PCR (BAL, PRW) PJ	CMV	HSV	PCR; Culture (blood, faeces, urine, ascites, swab)	TG IgG (AU/mL)
P11	171	abn	*N/A*	- (BAL)	- (BAL)	*N/A*	*N/A*	*S. epidermidis* (b)	-
P12	154	abn	*N/A*	*C. glabrata* (BAL)	- (BAL)	+ (BAL)	- (BAL)	*C. tropicalis* (b)<br>*C. tropicalis* (u)<br>*EBV reactivation* (b)	+ (22)

Table S3b: IRC B survived (n=5)

RCW and/or ImCU									
	CD4+ count/µL	R_xCI	N-ß-D-glucan (serum)	Culture (BAL, Sputum, Tracheal Fluid)	PCR (BAL, PRW, Sputum) PJ	CMV	HSV	PCR; Culture (blood, faeces, urine, ascites, swab)	TG IgG (AU/mL)
P10	77	abn	*N/A*	*K. pneumoniae* (Sp)<br>*St. maltophilia* (Sp)	- (Sp)	*N/A*		*K. pneumoniae* (b)<br>*S. epidermidis* (b)<br>*S. epidermidis* (a)<br>*C. glabrata* (f)	-
P13	183	abn	*N/A*	*M. catarrhalis* (Sp)<br>*Str. dysgalactiae subspecies equisimilis* (Sp)	+ (Sp) Ct: 31,7	*N/A*		*C. glabrata* (u)	-
P14	198	n	*N/A*	*N/A*	*N/A*			*E. coli* (b)	-
P15	188	n	*N/A*	*N/A*	*N/A*			*HEV-Infection* (b)	-
P16	102	n	523	*N/A*	*N/A*			*S. epidermidis* (b)<br>*E. faecium* (b)<br>*C. parapsilosis* (b)	-

Table S3a and S3b (IRC B). *EBV: Epstein–Barr virus. HEV: Hepatitis E virus. Levels of care: RCW (Regular Care Ward), ImCU (Intermediate Care Unit), ICU (Intensive Care Unit). Radiological chest imaging (R_xCI): n = normal; abn = abnormal. Sample sources: a = ascites; b = blood; Sp = sputum; s = swab; u = urine; f = faeces; TF = tracheal fluid. BAL: bronchoalveolar lavage. PRW: pharyngeal rinse water. PJ: Pneumocystis jirovecii. CMV: cytomegalovirus; HSV: herpes simplex virus. TG: Toxoplasma gondii. N/A: not applicable.*

Supplementary Information 1

Table S4a and S4b (IRC C). Required hospital care level (LHC) and in-hospital mortality (IHM) rates for participants within Immunological risk cluster C (IRC C).

Table S4a: IRC C died (n=3)

	CD4+ count/µL	R_xCI	N-ß-D-glucan (serum)	Culture (BAL)	PCR (BAL) PJ	CMV	HSV	Culture (blood, faeces, urine, ascites)	TG IgG (AU/mL)
**RCW and/or ImCU**									
P20	253	n	136	*N/A*	*N/A*			*C. albicans* (u)<br>*E. faecium* (u)<br>*E. coli* (a)<br>*Cryptosporidium sp* (f)<br>*S. epidermidis* (b)	+<br>(43)
**RCW and/or ImCU and ICU**									
P18	370	abn	*N/A*	Adenovirus	-	-	-	*E. faecalis* (u)<br>*S. epidermidis* (a)	-
P17	250	abn	*N/A*	*K. pneumoniae* (BAL)<br>*C. glabrata* (BAL)<br>*C. albicans* (BAL)<br>*E. faecium/VRE* (BAL)	+<br>Ct: 28,92	+	-	*S. epidermidis* (b)	-

Table S4b: IRC C survived (n=3)

	CD4+ count/µL	R_xCI	N-ß-D-glucan (serum)	Culture (BAL)	PCR (BAL) PJ	CMV	HSV	Culture (blood, faeces, urine, ascites)	TG IgG (AU/mL)
**RCW only**									
P21	471	n	*N/A*	*N/A*	*N/A*			*S. epidermidis* (a)<br>*E. faecium* (a)<br>*C. kefyr* (u)	+<br>(27)
**RCW and/or ImCU**									
P19	273	n	*N/A*	*N/A*	*N/A*			*Str. agalactiae* (u)	-
P22	237	n	*N/A*	*N/A*	*N/A*			-	-

Table S4a and S4b (IRC C). *Levels of care: RCW (Regular Care Ward), ImCU (Intermediate Care Unit), ICU (Intensive Care Unit). Radiological chest imaging (R_xCI): n = normal; abn = abnormal. Sample sources: a = ascites; b = blood; Sp = sputum; s = swab; u = urine; f = faeces; TF = tracheal fluid. BAL: bronchoalveolar lavage. PRW: pharyngeal rinse water. PJ: Pneumocystis jirovecii. CMV: cytomegalovirus; HSV: herpes simplex virus. TG: Toxoplasma gondii. N/A: not applicable.*

Common comment for Table S2a-b, S3a-b, S4a-b on Serum (1→3)-β-D-Glucan Testing:

In our study, 3 out of 22 patients with CD4<500/µl tested positive for PJ by PCR. However, serum β-D-glucan (BDG) testing was not performed for these patients. Among those, two cirrhotic patients—one in cluster IRC-A and one in IRC-C—had intermediate PCR cycle threshold (Ct) values of 27–30, suggesting a relatively low fungal burden; both survived without specific PJP therapy. The third patient, an HIV-positive individual with MASLD and a CD4 count of 183/µl, showed radiographic abnormalities and received trimethoprim–sulfamethoxazole (TMP–SMX) empirically, also without BDG testing. In the cirrhotic patients, extensive *Candida* colonization would likely have complicated the interpretation of BDG levels had they been available.

Although the absence of BDG measurements limits the ability to further characterize fungal burden—particularly in the two cases with intermediate Ct values—it does not materially affect our analysis, as both cirrhotics had favorable outcomes without PJP-directed treatment.

Supplementary Information 1

BDG was intended as an adjunct to molecular diagnostics: elevated BDG levels would have supported PJP in cases with intermediate Ct values, whereas indeterminate or negative BDG in combination with high Ct values would have argued for colonization. Selected serum samples were planned for duplicate measurement, with those exceeding 500 pg/mL to be diluted and reanalysed.

Supplementary Information 1

Table S5. Demographic characteristics, clinical and laboratory parameters, and Mortality Risk Stratification at admission to hospital of patients with liver disease (N = 15) and $CD4^+$ <200/µl or 200–499/µl assigned to IRC A (N = 9) or IRC C (N = 6), respectively. Of those, 3 were post Liver Transplantation and 12 with active liver cirrhosis.

					N (% of all 15 patients)
		**MD [Q1;Q3] , (range: min-max)**			**N (% of all 15 patients)**
**Age (y)**		53 [47; 63], (29 – 69)			
**Sex, n (%)**	♀				6 (40%) [4 (IRC A) + 2 (IRC C)]
	♂				9 (60%) [5 (IRC A) + 4 (IRC C)]
**Body Mass Index (kg/m²)**		25.7 [19.0; 26.3], (18.0 – 35.8)			
**Diseases (≠Liver Cirrhosis)**					
HIV/AIDS					0 (0%)
Post Liver Transplantation					3 (20%)

**Liver Cirrhosis**				**N (% of 12 patients)**
Cause:	Primary sclerosing cholangitis (PSC)			3 (25%)
	Alcoholic steatohepatitis (ASH)			5 (42%)
	Non-alcoholic steatohepatitis (NASH)			2 (17%)
	Cryptogenic cirrhosis			2 (17%)

Child-Pugh Score [Classification]	5-6	points	[stage A]	1 (8%)
	7-9	points	[stage B]	7 (58%)
	10-15	points	[stage C]	4 (34%)
**Risk Stratification at hospital admission**				
SAPS 3 Score	64 [61; 67], (29 – 76)			
SAPS 3 90-days mortality rate (%)	38 [32; 45], (1 – 63)			
CCI Score	6 [5;8], (3 – 10)			
CCI-Mortality-risk 10 years (%)	89 [78; 99], (22 – 100)			

**Laboratory values at hospital admission**	**MD [Q1;Q3] , (range: min-max)**	**Normal range**
aPTT (seconds)	30 (28; 35], (27 – 60)	24.4 – 32.4
INR	1.11 [1.05;1.86], (0.97 – 2.40)	
Fibrinogen (mg/dl)	218 [157; 345], (70 – 472)	180 – 350
Leucocytes (cells/nl)	9.1 [4.5; 12.1], (3.5 – 19.5)	4.0-10.0
Hemoglobin (g/dl)	9.2 [8.4; 9.9], (7.2 – 12.9)	13.7 – 17.2
Thrombocytes (cells/nl)	88 [68; 135], (34 – 255)	140 – 320
Creatinin (mg/dl)	1.5 (1.3; 3.2], (0.7 – 6.3)	0.9 – 1.3
Bilirubin total (mg/dl)	1.4 [1.1 ;2.3], (0,6; 14)	0.3 – 1.2
Albumin serum (g/dl)	2.9 [2.2; 3.0], (2.1 – 4.2)	3.4 – 4.8
CRP (mg/dl)	5.5 [2.7; 12.8], (1.2 – 30.5)	<0.5
pH	7.36 [7.31; 7.45], (7.21 – 7.48)	7.36 – 7.44

Table S5. *IRC = immunological risk cluster; HIV = human immunodeficiency virus; AIDS = acquired immunodeficiency syndrome; PSC = primary sclerosing cholangitis; ASH = alcoholic steatohepatitis; NASH = non-alcoholic steatohepatitis; SAPS 3 = Simplified Acute Physiology Score 3; CCI = Charlson Comorbidity Index; aPTT = activated partial thromboplastin time; INR = international normalized ratio; CRP = C-reactive protein.Continuous variables are presented as median [first quartile; third quartile] with range (minimum–maximum), while categorical variables are shown as absolute numbers and percentages, as appropriate. Liver disease etiology and Child–Pugh stages are reported for patients with established cirrhosis only. Risk stratification at hospital admission was assessed using the Simplified Acute Physiology Score 3 (SAPS 3), estimated 90-day SAPS 3 mortality, and the Charlson Comorbidity Index (CCI) with corresponding 10-year mortality risk. Laboratory parameters were obtained at the time of hospital admission and are reported together with reference ranges.*
