# Supplementary material for: Are CD4+ T-Cell Counts Associated with Pneumocystis jirovecii Detection in Hospitalized Patients with Liver Disease? A Retrospective Exploratory Pilot Analysis
Source: Livers. Author manuscript; Available in PMC 2026 Jun 2. (PMC13225858; doi:10.3390/livers6030040)
Supplement: supplemental 2 [file NIHMS2179768-supplement-supplemental_2.pdf]

Supplementary Information 2

Screening for *Pneumocystis jirovecii* (DNA): PJ detection was routinely performed using bronchoalveolar lavage (BAL) fluid samples if clinically indicated, via flexible bronchoscopy (FB) and/or sputum aspiration in selected cases. Following centrifugation at 4000 rpm for 10 minutes, samples were aliquoted for immediate molecular testing. DNA extraction was carried out using the QIAamp DNA Mini Kit (Qiagen GmbH, Hilden, Germany). Detection of *Pneumocystis jirovecii* DNA was conducted via real-time PCR using the *Pneumocystis jirovecii (carinii)* Real-TM® assay (Sacace Biotechnologies Srl, Como, Italy), targeting the 26S rDNA, as described previously [1]. Amplification was performed on a CFX96 Touch Real-Time PCR Detection System (Bio-Rad Laboratories, Hercules, CA, USA), including internal (β-globin gene) and external positive/negative controls. Samples were considered positive when cycle threshold (Ct) values were ≤38 and exhibited a characteristic sigmoidal amplification curve.
To estimate fungal burden:

- Ct <27 was considered high fungal load and strongly associated with microscopically confirmed PJP.
- Ct 27–30 indicated intermediate burden requiring clinical correlation.
- Ct >30 was interpreted cautiously as potential colonization.

Quantification of β-D-Glucan: For a subset of patients, serum samples were tested for (1→3)-β-D-glucan (BDG) using the Fungitell® assay (Associates of Cape Cod, East Falmouth, MA, USA) according to the manufacturer's instructions. The assay was conducted using a commercially available kit that meets quality standards in accordance with CE certification requirements. Quantification was performed via a kinetic reader, and values were compared with standardized reference curves [2]. Results were interpreted as:

- < 60 pg/mL: negative
- 60 – 80 pg/mL: indeterminate
- > 80 pg/mL: positive

References

1. Rath, P.-M. and J. Steinmann, *Update on Diagnosis of Pneumocystis Pulmonary Infections.* Current Fungal Infection Reports, 2014. **8**(3): p. 227–234.
2. Damiani, C., et al., *Combined Quantification of Pulmonary Pneumocystis jirovecii DNA and Serum (1→3)-β-d-Glucan for Differential Diagnosis of Pneumocystis Pneumonia and Pneumocystis Colonization.* Journal of Clinical Microbiology, 2013. **51**(10): p. 3380–3388.
